# Patient and staff acceptability of an artificial intelligence software to prioritize chest X-rays in an NHS setting: a qualitative evaluation

**DOI:** 10.1186/s12913-026-14950-z

**Published:** 2026-06-18

**Authors:** Emma J. Steel, Sean F. Duncan, Mark Hall, John D. Maclay, David B. Stobo, Benjamin Gregory, David J. Lowe, Evi Germeni

**Affiliations:** 1 Health Economics and Health Technology Assessment (HEHTA), School of Health and Wellbeing, University of Glasgow, Clarice Pears Building, 90 Byres Road, Glasgow, G12 8TB UK; 2 Digital Health Validation Lab, College of Medical, Veterinary and Life Sciences, University of Glasgow, Glasgow, UK; 3 Radiology, Queen Elizabeth University Hospital, Glasgow, UK; 4 Respiratory Medicine, Glasgow Royal Infirmary, Glasgow, UK; 5 School of Medicine, Dentistry and Nursing, University of Glasgow, Glasgow, UK; 6 Emergency Department, Queen Elizabeth University Hospital, Glasgow, UK

**Keywords:** Artificial intelligence, Chest X-rays, Lung cancer, Theoretical framework of acceptability, Longitudinal qualitative evaluation, Focus groups, Interviews, Health technology assessment, Clinician perspectives, Patient perspectives

## Abstract

**Background:**

Chest X-rays (CXRs) remain the first-line investigation in the lung cancer (LC) diagnostic pathway; however, the time from the original referral for CXR to computed tomography (CT) and respiratory review can currently take several weeks for outpatients. Qure.ai’s ‘qXR’ software can be utilized to speed up the detection of LC by flagging abnormalities in CXRs for priority reporting, has the potential to enable outpatient CT imaging within 72 h of CXR and reduce the time from referral to reported CT by several weeks. A qualitative evaluation with a longitudinal aspect was designed to provide an in-depth understanding of the acceptability and behavioral impact of the software from the perspectives of both NHS staff and service users.

**Methods:**

Repeat, semi-structured interviews were conducted with 16 NHS staff (radiologists, radiographers, and administrators) before and after implementation of the software in a Scottish NHS board. Eight NHS service users participated in focus groups to explore their experiences and attitudes toward the software. The Theoretical Framework of Acceptability informed data collection; however, the data analysis employed an inductive thematic approach.

**Results:**

Staff viewed the software as a useful adjunct and believed it could expedite LC pathways; service users perceived the potential for better care through earlier diagnosis and an accelerated pathway to treatment. The radiology staff raised concerns about the software’s accuracy and interpretations, as well as the potential for complacency and biased decision-making. Staff perceived that the change in the patient pathway performed better than anticipated, and service users voiced a preference for short-term anxiety with prompt action (AI pathway) to prolonged uncertainty (standard pathway).

**Conclusions:**

This study identified key facilitators of and barriers to the acceptability of AI software for prioritizing CXRs. There is support for AI-assisted CXR assessment, provided that it improves patient outcomes and is used in conjunction with clinicians. A hierarchy of confidence in the software’s performance significantly impacted the experienced acceptability of the technology. To support stable, long-term adoption, AI software must integrate easily into existing systems, perform accurately, and not disrupt practice or negatively impact patient pathways.

**Supplementary Information:**

The online version contains supplementary material available at https://doi.org/10.1186/s12913-026-14950-z.

## Introduction

Lung cancer (LC) is the third most common cancer in the UK and the most common cause of cancer death [1]. Early diagnosis and reduced time to treatment are associated with improved five-year survival rates for patients with lung cancer [2]. The NHS Scotland Optimal Lung Cancer Diagnostic Pathway aims to achieve diagnosis by day 21 and treatment by day 42 [3, 4], which is an ambitious target compared with the 62-day UK standard from receipt of referral to treatment [5]. This target has not yet been widely achieved, with only 80% of LC patients in Scotland being treated within the 62-day standard in early 2025 [6].

Chest X-ray (CXR) is the most commonly used initial imaging test for evaluating possible lung cancer in clinical practice; however, the time for outpatients from original referral for CXR to computed tomography (CT) and respiratory review is variable and can take several weeks [2, 7, 8]. The artificial intelligence (AI) software, qXR, is a comprehensive AI solution for CXR interpretation [9]. With the ability to assess CXRs within 30 seconds of acquisition, the software can be utilized by radiology staff to prioritize CXR reporting based on the presence or absence of LC features, flagging urgent suspected cancers (USCs) for priority reporting. Implementing qXR in the LC diagnostic pathway has the potential to improve the accuracy and efficiency of reporting CXRs, enable CT imaging within 72 h of CXR, and, in turn, reduce the time from referral to reported CT by several weeks [10].

To assess the clinical effectiveness and acceptability of the AI software to prioritize CXR interpretation, a stepped-wedge cluster-randomized study with embedded qualitative research was conducted over a 12-month period in Glasgow, Scotland (RADICAL) [10]. The qualitative evaluation sought to provide an in-depth understanding of the acceptability and behavioral impact of the software among key stakeholders, namely, NHS staff and service users. Acceptability has become a key consideration in the design, implementation and evaluation of healthcare interventions and technologies. If an intervention is considered acceptable, healthcare professionals are more likely to engage with it and deliver it as planned [11]. Service users are also more likely to be satisfied with their treatment and benefit from improved outcomes [12, 13]. In addition, NICE have identified the importance of employing qualitative methodologies in the context of health technology assessment (HTA) [14], and there is recognition that rigorous qualitative studies, which systematically capture the voices of key stakeholders, can provide crucial high-quality evidence to complement quantitative findings [15, 16].

## Methods

### Study design

To understand both the anticipated and experienced acceptability of the software, a longitudinal descriptive qualitative study was conducted, based on repeat, semi-structured interviews with NHS staff (i.e., radiologists, radiographers, and hospital administrators working in the radiology department) before and after implementation. To capture the NHS service user experience, focus groups were conducted with individuals who had a CXR performed at one of the participating hospitals. Extensive patient and public involvement and engagement were undertaken during the study design phase.

This study drew on Sekhon et al.’s Theoretical Framework of Acceptability (TFA) [17], which constitutes the first effort to define ‘acceptability’ systematically while offering seven conceptually distinct constructs to capture its key dimensions (i.e., affective attitude, burden, ethicality, intervention coherence, opportunity costs, perceived effectiveness, self-efficacy). A key feature of the TFA is its incorporation of a temporal perspective, distinguishing between prospective and retrospective acceptability.

### Participants

The study was set within NHS Greater Glasgow and Clyde (GGC); the health board was split into three sectors, comprising nine hospitals where over 76,000 outpatient CXRs are conducted annually [10]. The three sectors implemented the software in a stepped-wedge manner at 2-month intervals [18]. At each sector, radiology staff were invited to attend an information session about the software’s implementation; they were also informed about the qualitative aspect of the study and invited to contact one of the study’s Research Assistants (ES and BG) if they were interested in participating. Further reminders about the qualitative study were emailed from the Chief Investigator, and those who took part in an initial interview were asked by the Research Assistants to help recruit colleagues who might be interested in participating. The inclusion criteria for NHS staff were as follows: radiographers, radiologists and hospital administrators working in the radiology department at one of the participating sites. Those who agreed to be contacted about the qualitative study were emailed copies of the participant information sheet and consent form before participating in an online interview.

Once the software had been implemented in the third sector for 4 + months, eligible NHS service users were mailed copies of the participant information and consent form and asked to contact ES if they wished to participate in a focus group. The inclusion criteria for NHS service users were as follows: over 18 years old with posterior anterior chest radiograph, acquired consecutively during usual care through the outpatient referral pathway, including general practitioners (GPs), whose radiograph was assessed by the software. The focus groups were not restricted to individual sectors; they were a mixed cohort of all three sectors.

### Data collection

NHS staff interviews were conducted online (via MS Teams) [19] prior to the implementation of the software and again 5 + months post-implementation. The interviews lasted, on average, 45 min pre-implementation and 30 min post-implementation. Staff interviews were conducted by ES and BG, who have substantial training and experience in qualitative data collection and analysis; ES has a healthcare background, and BG is a trained sociologist. NHS service user focus groups were conducted either online (via MS Teams) or in person, based on participants’ preference, by ES, 4 + months post-implementation in the third sector. Focus groups were segmented as follows: Group 1 included service users who had a CT performed following an abnormal CXR and were diagnosed with LC; Group 2 included service users who had a normal CXR and did not have a CT performed. The focus groups lasted an average of 60 min. The interview and focus group guides were developed based on the key constructs of the TFA and focused on the study objectives (Table 1; the full interview and focus group schedules are available as supplementary files).

**Table 1:** Key interview/focus group questions based on the TFA constructs

TFA construct	Questions
Affective attitude	What were your first thoughts when you heard about the introduction of the qXR software?
Intervention coherence	What is your understanding of the software and how it works?
Burden and opportunity costs	What kind of changes are needed for the implementation of the software? *(for staff interviews)* What are the positive and what are the negative aspects of introducing this AI technology in the NHS? *(for patient focus groups)*
Ethicality	There is a lot of discussion about the ethical and legal challenges of using AI technologies in health care. What is your view on this?
Perceived effectiveness	To what extent do you think that the qXR software will be effective in reducing delays to obtaining a CT scan, when necessary, after chest X-ray?
Self-efficacy	Overall, how confident do you feel in using the qXR software? *(for staff interviews)*

### Data analysis

With the participants’ consent, the online interviews and one online focus group were recorded and transcribed verbatim using MS Team’s built in recording and transcription features [19]; the two in-person focus groups were audio-recorded using a Dictaphone, and transcribed verbatim using the Otter.ai online transcription feature [20]. All transcripts were de-identified and checked for accuracy by ES and BG. QSR NVivo 14 data management software was employed for the coding of the transcripts [21]. While the TFA informed data collection, data analysis employed an inductive thematic approach, identifying themes from participants’ expressed perspectives [22]. The coding framework was discussed iteratively by ES and EG as it developed, with potential interpretive bias identified and addressed to enhance clarity. To capture each individual’s temporal perspective, the data from the NHS staff interviews were summarized into individual pen portraits [23]. To capture the NHS service user perspective, focus group data were summarized into representative pen portraits of a group [24]. Pen portraits were designed by Sheard and Marsh [23] to analyze longitudinal qualitative data from multiple sources without losing the richness of the data (Appendix 1).

## Results

### Participants

Sixteen NHS staff members completed a pre-implementation interview, and 14 of them participated in a post-implementation interview (Table 2). Eight NHS service users participated across three segmented focus group discussions (Group 1: *n* = 2 (one in-person focus group); Group 2: *n* = 6 (one in-person focus group (*n* = 3) and one online focus group (*n* = 3)).

**Table 2:** Participant characteristics

NHS staff demographics			
		Pre-implementation (n=16)	Post-implementation (n=14)
Gender	Female	12	10
	Male	4	4
Job role	Radiologist	8	7
	Radiographer	6	5
	Administration	2	2
Glasgow sector	1	5	3
	2	5	5
	3	6	6
**Service user demographics** (n=8)			
Age	45-54 years		2
	55-64 years		1
	65-74 years		1
	75-84 years		3
	85+ years		1
Gender	Female		5
	Male		3
Highest level of education	High school or leaving certificate		4
	Certificate or diploma		1
	University degree or higher		3
Current employment status	Unemployed		1
	Paid employment (full- or part-time)		1
	Retired		4
	Unable to work (sickness/disability)		1
	Doing unpaid or voluntary work		1
Lung cancer diagnosis	Yes		2
	No		6

### A useful adjunct to speed up the reporting of USCs

Across the board, the participating staff deemed the current CXR reporting process to be unfit, overwhelming, and unsustainable in a climate of unprecedented demand and diminishing workforce resources (Table 3). Before implementation, staff hoped that the software would successfully shorten the time from CXR to reported CT. Post-implementation, they deemed that the software had largely achieved this purpose, with qualifying patients accessing CT within a few days and fewer USCs found in the backlog of CXRs. Service users’ experiences echoed this; some Group 2 participants waited several weeks to receive their normal CXR result. Conversely, both Group 1 participants who were diagnosed with LC experienced an accelerated pathway and had a CT performed within a few days of their CXR: *“CT scan within 3 to 4 days*,* treatment within 3 weeks… My experience has been first class with it… I feel lucky that I’ve been diagnosed at a time that they’re doing this.”* (Service user #1, Group 1).

Some of the participating staff’s colleagues were initially concerned that the introduction of AI technology could threaten jobs, and there was a *“background level of scepticism”* (Radiologist #1, pre-implementation) regarding the software. While some of them reportedly became more engaged with the software during implementation, others refused to interact with it, and it was recognized that *“there’s always gonna be those people*,* no matter how much we’re trying to explain it to them*,* this isn’t a system that’s going to report chest X-rays for us. This is a tool to help our workflow”* (Radiologist #1, post-implementation). Similarly, while service users hoped that the software could improve efficiency and accuracy, they also considered the balance between freeing up NHS staff time and preserving human input.

NHS staff and service users were unanimous in viewing the software as a workflow tool or a useful adjunct to the diagnostic pathway, and they felt reassured that it was not working autonomously. Service users also perceived a potential for better service, one that they would travel for if needed, with human-AI collaboration providing *“a more accurate picture”* (Service user #8, Group 2) and *“a better chance of survival”* (Service user #7, Group 2).

**Table 3:** Additional illustrative quotes from staff interviews and service user focus groups

A useful adjunct to speed up reporting of USCs	
**Shortening the time from CXR to reported CT**	
	*“We know there will be patients sitting in our backlog that have serious pathology. Absolutely anything we can do to tease those out and deal with them quicker will absolutely improve patient care.”* (Radiologist #1, pre-implementation)
	*“I definitely think there’s a faster turnaround for the chest X-rays that have something on them…Historically*,* my heart would have sunk if you came across a chest X-ray that was a couple of weeks old*,* or older. And you think*,* there’s definitely something suspicious on that hilar. They need a CT. And then it’s another couple of weeks before they get the CT.”* (Radiologist #5, post-implementation)
***A workflow tool or useful adjunct to the diagnostic pathway***	
	*“I don’t think it can work in isolation. I’ve not found a cohort of patients I’m confident with that it can replace reporting. I think there’s always subtleties there which the GPs or referrers need to know about.”* (Radiologist #6, post-implementation) *“It’s not caused any delays to the patients coming through the department. And it’s been interesting to see what it’s flagging up at the time of image acquisition as well*,* so it kind of encourages you to look more at your image and say*,* oh yeah*,* it’s highlighted this as well… I think it’s already encouraging radiographers to look more for pathology.”* (Radiographer #5, post-implementation). *“I like the two working together*,* I feel like one’s given the other one back up. But the idea of no clinical input*,* I wouldn’t feel as confident and it would unnerve me*,* I think*,* if I thought it was all on its own.”* (Service user #8, Group 2) *“I would feel the same way. I would feel a bit nervous about AI not picking it up. I would always want the clinician giving his input.”* (Service user #4, Group 2)
**Acceptability of the software’s accuracy**	
***Accepting of the level of sensitivity***	
	*“It is over-calling*,* which means that it’s generating a lot of reports that don’t necessarily have anything on them. But I guess that’s just the flip side. I think*,* you know for its purpose*,* it seems to be doing the job.”* (Radiologist #1, post-implementation)
***Unaccepting of the level of sensitivity***	
	*“In young people*,* I find that it’s sometimes overcalled the hilar… Those ones I’ve definitely saved and I’ve gone and got a second pair of eyes on them. Only because I’ve had AI say those hilar are abnormal. But if I’d reported that myself with no input from AI*,* I would have called it normal and just carried on.”* (Radiologist #5, post-implementation)
***Accepting of the level of specificity***	
	*“There have been instances where things haven’t been picked up that were positive when you go back and review*,* but I suppose that’s part of a trial*,* things are not gonna be 100% accurate… It wasn’t something that was totally sticking out*,* glaring out at your face*,* but if you had appraised the image then you could have seen it.”* (Radiographer #4, post-implementation)
***Unaccepting of the level of specificity***	
	*“If the AI is not picking up smaller and more subtle abnormalities*,* people might be falsely reassured and just dismiss the X-ray as normal when it’s not.”* (Radiologist #4, pre-implementation) *“I have had several cancers I’ve found that it hasn’t*,* which worried me… On a few occasions*,* what it said is normal has shown a significant lung nodule. That worries me.”* (Radiologist #6, post-implementation)
***The software needs to be more accurate to be useful across the board***	
	*“I think it’s useful for most people already. For me to think it was useful to all I’d love to go through a 2–3 month period where it was picking up cancers that I wasn’t. And then I would be really happy.”* (Radiologist #6, post-implementation) *“I’ve seen a couple of occasions when people have organized a CT off the basis of the report when they haven’t spent enough time looking at it themselves… It’s just a confidence thing*,* because they’re not seeing as many [chest X-rays].”* (Radiologist #6, post-implementation)
**Acceptability of changes to the patient pathway**	
***Potentially anxiety-inducing for patients***	
	*“I think clinical communication has to come from someone who’s trained in clinical communication. And I don’t think it’s appropriate at all for admin staff to be contacting patients about a potentially anxiety-inducing process.”* (Radiologist #7, pre-implementation) *“GPs are supposed to pre-warn them. I can tell you right now that will not happen… And we’re gonna get asked a thousand and one questions*,* terrifying the life out of a patient because they’re having to come back for a CT scan without having any*,* no. I’ve got grave concerns on this and I wouldn’t be happy to phone the patient.”* (Administration #1, pre-implementation)
***Small number of patients found the new pathway anxiety-inducing***	
	*“About probably 10% of the patients had that panic phone call to the office to say*,* why am I being recalled?. It’s just the odd one with complicated questions that’s been bounced to somebody else to follow-up.”* (Radiographer #4, post-implementation)
***Accepting of new pathway***	
	*“I would think most patients want speed. Something’s wrong*,* what’s the quickest way that I can find out and get this dealt with? I would think even if you have a couple of days of anxiety*,* you can get seen quickly*,* and you would think that’s been a brilliant service.”* (Service user #8, Group 2)
**The software’s impact on workload and workflow** ***Potentially negative impact on workload and workflow***	
	*“They’re just worried that they’ll have to leave their room*,* where they’ve got more patients waiting in the cubicles. Then go and find somebody… That’s probably the main concern I’m getting just now.”* (Radiographer #3, pre-implementation)
***Impact was nil or minimal***	
	*“So the reporting room’s just around the corner and just being able to drop it into a box without interrupting anyone or waiting for someone to come off the phone is a good way of doing it… If we’d seen something [before]*,* we’d approach the duty radiologist who is constantly dealing with a million queries. So in a way*,* maybe it’s faster.”* (Radiographer #5, post-implementation)

### Acceptability of the software’s accuracy

Pre-implementation, most participating staff expected the software to be overly sensitive (i.e. too many false positives), and some were hopeful that it might increase LC detection. Participating radiologists and radiographers predicted that they would accept a level of sensitivity, and they voiced a preference for false positives over false negatives (Table 3). Post-implementation, they reported that the software was overly sensitive; however, few considered this level of sensitivity acceptable, and some reported issues with the software’s categorization of abnormalities. While the participating radiologists became confident in disagreeing with the software’s interpretations, they raised concerns about the potential for biased reporting among less experienced radiologists.

With respect to the software’s specificity, a few of the participating radiologists were concerned about the impact of false negatives, both pre- and post-implementation. Although CXRs not flagged by the software would still be viewed by a reporting radiologist under the standard pathway, consideration was given to whether subsequent complacent reporting could lead to these USCs ultimately being missed. Overall, the participating radiologists felt that the software needed to be more accurate to be useful across the board. One experienced radiologist shared a desire for the software to perform *“better than the humans”* (Radiologist #6, pre-implementation).

Despite not wanting the software to work autonomously, service users had high expectations for the software’s accuracy, with many also expecting it to be better than a human. There were mixed responses concerning whether service users would be more accepting of human or AI errors. Some imagined that they would feel *“quite cheated”* (Service user #3, Group 2) and disappointed if the software missed a USC on their CXR. Others felt that while they would not benefit from the software’s accelerated pathway in this scenario, it would not worsen their situation compared with the standard pathway: *“At the moment we’re in its infancy. I don’t see that that’s really an issue if it misses one. Okay*,* if it was me*,* I wouldn’t be happy*,* but your doctor misses things.”* (Service user #1, Group 1).

### Acceptability of changes to the patient pathway

Before implementation, the primary concern among the participating staff was that the change in the patient pathway may induce anxiety in patients and place extra pressure on administrative staff (Table 3). Under the standard pathway, CXR results are typically communicated to patients by their referring clinician (e.g., their GP), who then requests a CT scan and respiratory review for USC cases. Under the qXR pathway, USC cases were instead able to be contacted directly by hospital administration staff. Before implementation, the participating administrative staff members viewed contacting USC patients for an urgent CT scan as outside their remit.

Post-implementation, the radiology staff perceived that the qXR pathway had performed better than expected, that the administrative staff had adjusted well, and that most patients had responded positively. At the beginning of implementation, a small number of patients reportedly found the qXR pathway anxiety-inducing. Following some phone calls from concerned patients, GPs within GGC were sent a reminder to advise CXR patients about the possibility of an immediate CT scan. A subsequent decrease in phone calls from concerned patients led to the speculation that *“patients were obviously more aware”* (Radiographer #4, post-implementation).

One of the administrative participants disclosed that she and some of her team chose not to participate in the study. While she supported the software’s purpose, she maintained that contacting USC patients for an urgent CT was outside their remit. Although she had not spoken to any patients herself, she was aware that *“the staff did say the patients were fine. But then you’ve got other patients who are not fine. Then they want to ask the questions”* (Administration #1, post-implementation). By comparison, the other administrative participant had a *“mostly very positive experience”* (Administration #2, post-implementation) despite her initial concerns, with only one of the estimated 20 patients she contacted asking questions that required a clinician’s attention.

The Group 1 service users were contacted by a clinician to attend for an urgent CT; however, they did not think they would have had an issue with hospital administration contacting them. Similarly, some of the Group 2 service users stated that they would be *“happy with it”* (Service user #5, Group 2) and *“it wouldn’t worry me”* (Service user #4, Group 2). Others recognized that while being unable to initially speak to a clinician about their CXR results would likely induce anxiety, they would prefer short-term anxiety with prompt action (AI pathway) over prolonged uncertainty (standard pathway). Before receiving a letter about the qualitative study, none of the service users were aware that software was used to analyze their CXR. While service users did not think it was necessary to inform patients about the software, overall, they and the participating staff felt that GPs needed to inform patients about the change to the patient pathway and the possibility of an immediate CT: *“I don’t see that that’s not beneficial*,* as in*,* allaying people’s fears”* (Service user #1, Group 1).

### The software’s impact on workload and workflow

Pre-implementation, most participating radiologists and radiographers expected the impact on workload to be minimal. Some of the participating radiologists, however, did not have time set in their schedules to report CXRs and had to specifically organize time to engage with the software during its implementation. There was also concern among some participating radiographers about whether the software would affect their workflow, especially if they had to take time to manage patient anxieties or track down a radiologist to report a USC. The impact on the administrative staff’s workload was also considered potentially negative, given ongoing inadequate staffing levels.

Post-implementation, most participants reported that the impact on their workload and workflow had been nil or minimal. Each site developed a process to alert radiologists to flagged images. From the perspective of the participating radiographers, the new processes were more streamlined and less disruptive than their usual processes were.

However, three of the participating radiologists, all of whom regularly reported CXRs, mentioned that the software increased their reading times. The software’s level of sensitivity meant that they questioned its interpretation each time, even though *“90 odd percent of the time it’s not something that’s significant…might make I report 1400 per week as opposed to 1500”* (Radiologist #6, post-implementation). One also found comparing the AI image to the current and previous CXRs to have *“significantly increased”* their reading times because of the time taken to manipulate the images on their screen and *“very frustrating”* that the software could not access previous images, as *“the vast majority of these patients have got previous CXRs”* (Radiologist #4, post-implementation).

### Liability

Pre-implementation, liability for radiologists was raised as the primary ethical and legal concern and was reportedly *“making a few people feel slightly vulnerable”* (Radiologist #1, pre-implementation). Post-implementation, while some of the participating radiologists were initially slightly wary of disagreeing with the software, they soon became comfortable overriding its interpretations and gained confidence that liability would not be any more of an issue than it currently is. It was considered that, while the software looked at CXRs in isolation, the radiologists had access to the full picture of their clinical history and previous images, and *“you’ve got clinical acumen that you lean into when you report*,* you’re not just a binary normal abnormal*,* there’s more to it”* (Radiologist #5, post-implementation).

Service users did not believe that liability would be an issue at this stage of development. Nevertheless, they anticipated that patients would be less tolerant of errors in the future when expectations of its accuracy are likely to be higher as *“the longer it’s in*,* the more confident we’ll become”* (Service user #5, Group 2).

### Overall confidence in the software

Pre-implementation, all the participating staff members felt confident about using the software, with an overall expectation that it would be *“pretty user friendly”* (Radiologist #5, pre-implementation) and *“pretty accurate”* (Radiologist #4, pre-implementation). Post-implementation, a distinction emerged between staff’s confidence in using the software and their confidence in its performance. While they all continued to feel confident in their ability to use the software and found it to be *“idiot proof in terms of how you access it”* (Radiologist #8, post-implementation), three of the radiologists completely lacked confidence in its ability to accurately detect signs of LC. One felt *“confident in using it*,* yeah*,* 100%*,* but confidence in its ability is very low”* (Radiologist #7, post-implementation).

Service users were confident in the software’s ability to reduce delays to CT when used in conjunction with clinicians, and overall they were optimistic about the future of AI in improving healthcare outcomes.

## Discussion

This study provides the first longitudinal, qualitative insight into the implementation of AI in CXR interpretation for the detection of LC, incorporating both NHS staff and service user perspectives, and highlights considerations for the future implementation of AI technology in this area. Our findings support Sekhon et al.’s view that the acceptability of healthcare interventions is a dynamic rather than a fixed construct shaped by ongoing interaction, experience, and contextual factors [17]. A binary assessment of acceptability would have missed critical concerns that affected the acceptability of the software among key stakeholders.

Overall, the software was viewed as a valuable workflow tool that improved prioritization and reduced delays to CT for USCs. While the software in this setting aimed to flag USCs for immediate reporting rather than increasing detection, concerns were raised about its accuracy and interpretations. Although most participating radiologists and radiographers anticipated that they would accept a degree of sensitivity post-implementation, many considered the software overly sensitive, and some often disagreed with its categorization of abnormalities. There was also evidence of a hierarchy of confidence in the software’s performance, with those who regularly report a large volume of CXRs often questioning its added value, which significantly affected their experienced acceptability of the technology. Blake et al. deployed qXR in an Emergency Department setting, aiming to improve the accuracy of initial CXR interpretation without waiting for a radiologist’s report [25]. They recognized that the *“software may work well but won’t provide any benefit to patients if staff aren’t engaged and using it routinely.”* Indeed, while the qXR algorithm reports high sensitivity and specificity for identifying abnormalities [26, 27], our qualitative insights present a more complex picture in which staff perceptions and trust in the tool play a critical role in shaping its real-world value and impact on patient care. Varying levels of trust in the software’s outputs are likely to influence its routine use outside a research setting. An evaluation of the software’s technical performance, along with other outcome measures such as the safety (false-negative rate) of the software in ruling out patients from entry onto the cancer pathway, will be reported in a separate publication.

While user complacency and biased decision-making were of notable concern both pre- and post-implementation, a study on the use of AI software to identify pneumothoraces on CXRs revealed that it actually benefited less experienced clinicians in particular and improved their diagnostic confidence and sensitivity [28]. Additionally, service users presented a dichotomy: they expected the AI to be highly accurate and better than a human but did not want it to work autonomously. This echoes findings from other studies, in which the majority of patients reported wanting AI in radiology to be equal to or more accurate than a radiologist, but with a strong preference for human clinician involvement and for AI to serve as a support tool [29–31]. While the radiologists in our study initially expressed liability concerns, they gained confidence, as they recognized the value of clinical judgment and contextual knowledge over AI interpretations. Moreover, both staff and service users supported the software’s use as an adjunct, not a replacement, for clinical decision making, reinforcing the view that AI in radiology should be positioned as a collaborative tool, complementing rather than substituting for human expertise.

Burden and workflow integration are both key factors underpinning acceptability [32]. For most participating staff, workflow was not negatively impacted. However, the radiologists who most frequently reported CXRs found the software’s level of sensitivity to be burdensome, significantly increasing their usual reading times and, in turn, reducing the number of images they could normally report. Furthermore, initial concerns over changes to the patient pathway were progressively resolved by staff, and service users confirmed that they would prefer short-term anxiety with prompt action (AI pathway) over long-term uncertainty (standard pathway). Establishing clear integration pathways is essential to ensure that AI tools complement existing clinical workflows without introducing disruption or ambiguity.

The temporal perspective of this study, captured through the TFA, was particularly helpful in demonstrating that several initial concerns (i.e., liability; changes to the patient pathway; workflow; perceived threats to jobs or professional roles) were largely alleviated over time, whereas others (i.e., level of sensitivity; accuracy of interpretations) proved more problematic than originally anticipated. Chen et al. explored the pre-adoption state of knowledge, awareness and attitudes toward AI among radiology staff [33] and suggested that the adoption of AI in this setting depends on the degree to which radiology staff *“engage positively and knowledgeably”* with it. To add to this, our findings highlight how when radiology staff engaged with the AI software, encountering both its benefits and limitations, their affective attitudes, perceived burden, ethical alignment, and confidence in the software shifted over time. Proof that judgments made at the point of introduction can therefore not be presumed to remain stable and that sustained engagement can shape not only attitudes but also the conditions under which AI is viewed as feasible, trustworthy, and aligned with professional values. One of the main barriers to implementing AI in a radiology setting will be engaging those with a *“background level of scepticism”* who are not willing to familiarize themselves with the technology. While some of the participants’ colleagues initially viewed AI as a threat to their jobs and several refused to interact with it at all, others reportedly became more engaged with the software during implementation, despite their initial reluctance. A lack of early involvement limits opportunities for hands-on exposure, which appears to be necessary for acceptability to evolve.

## Conclusion

Our findings highlight the need to go beyond technical validation and ensure that AI tools in radiology align with end users’ needs and practices. The successful implementation of AI software in CXR interpretation relies not only on algorithmic performance but also on the ongoing negotiation of trust, value, and workflow fit among those who use it (staff) and those affected by it (service users). Currently, ensuring that AI functions as an adjunct to, and not a replacement for, clinical expertise will remain central to achieving both professional confidence and public acceptability of AI use in this setting. To support stable, long-term adoption, implementation efforts must prioritize sustained engagement, transparent communication about AI capabilities and limitations, and thoughtful integration into existing pathways.

### Study limitations

Recruiting NHS staff and service users was challenging. Two staff interviewees were unable to participate in the second interview because they had changed roles before implementation. Several service users expressed interest in the study but did not feel well enough to participate, including those who had a CT performed but were not diagnosed with LC, resulting in a smaller service user sample size than initially intended. Given these difficulties, we believe that allowing participants to choose between an online or in-person focus group benefited recruitment; however, we acknowledge the different nature of the data produced, which may have influenced the comparability and richness of the findings. Additionally, although we are aware that some staff members refused to interact with the software, only one such staff member volunteered to participate in the qualitative study. A maximum variation sampling strategy could have encouraged a larger, more diverse sample and provided a wider range of perspectives. Finally, while the staff interviews followed the temporality that is useful in the context of the TFA, the service user focus groups did not. Interviewing service users before their CXR may have given us further insights.

## Appendix 1: Four key stages of the pen portrait process

**Table:** 

Key stage	NHS staff interviews (individual participant pen portraits)	NHS service user focus groups (representative pen portraits of a group)
1. Understand and define what to focus on	To understand both anticipated and experienced acceptability of the qXR software before and after implementation	To capture the NHS service user perspective
2. Design a basic structure relevant to the dataset in question	Thematic analysis and overall summaries for each NHS staff member	Thematic analysis and overall summaries for each group of NHS service users
3. Populate the content	Tables were populated for each individual with themes, overall summaries, and relevant quotes to illustrate their temporal perspective	Tables were populated for each of the groups with themes, overall summaries, and relevant quotes to illustrate the NHS service user perspectives
4. Interpretation	Through the development of a framework matrix, individual pen portraits were compared across sites, time points and against representative pen portraits from the NHS service user groups. The framework matrix was discussed by EG and ES to develop explanations and conceptual ideas of what was occurring in the data	

## Supplementary Information

Below is the link to the electronic supplementary material.


Supplementary Material 1


## Data Availability

The datasets generated during the current study are not publicly available due to human research ethics conditions, but are available from the corresponding author on reasonable request.

## Abbreviations

AI: Artificial intelligence
CT: Computed tomography
CXR: Chest X-ray
GGC: Greater Glasgow and Clyde
GP: General practitioner
HTA: Health technology assessment
LC: Lung cancer
TFA: Theoretical Framework of Acceptability
USC: Urgent suspected cancer
