# Supplementary material for: Patient and staff acceptability of an artificial intelligence software to prioritize chest X-rays in an NHS setting: a qualitative evaluation
Source: BMC Health Serv Res. 2026 Jun 18;26:1206. doi: 10.1186/s12913-026-14950-z (PMC13527979; doi:10.1186/s12913-026-14950-z)
Supplement: Supplementary file 1 [file 12913_2026_14950_MOESM1_ESM.pdf]

**PRE-IMPLEMENTATION STAFF INTERVIEW GUIDE**

**Prospective Acceptability of the qXR Artificial Intelligence Software**

| Introduction |
|---|
| Thank you for making the time to talk to me today. I understand how busy your schedule is, so I will aim to be finished within 45 minutes. As you know already, we are conducting interviews with staff, to better understand their views about the introduction of the **qXR** Artificial Intelligence Software. We plan to conduct two interviews with each participant, one now and the second one following implementation of the software.<br><br>All of the feedback you give me today will remain strictly confidential. Your name will not be attached to any of your comments, and any other potentially identifying information will be deleted or edited out from the transcriptions, to make sure that what you tell us remains private. Do you have any questions or is there anything else you would like to know before we start?<br><br>I have a list of key questions that I would like to ask you, but you may also have some information or ideas that we haven't thought of. I really want to hear what you think is important about this topic, so please feel free to tell me whatever you think relevant. I would like to record today's interview, so I can just concentrate on our conversation. Is that ok with you? Are you happy to get started?<br><br>**1. First of all, could you tell me a bit about yourself?**<br><br>*Possible follow-up questions:*<br>• How long have you been working at this hospital?<br>• What would you describe as the main challenges in your job?<br>• To what extent do you already use systems/tools to support you in diagnosis?<br>• What has been your experience so far with AI technologies? |
| Affective Attitude |
| *Purpose: To understand how participants feel about the technology.*<br><br>**2. What were your first thoughts when you heard about the introduction of the qXR Artificial Intelligence Software?**<br><br>*Possible follow-up questions:*<br>• How were you informed about the software?<br>• How clear did you find the information provided?<br>• How do you think your colleagues feel about the introduction of the software?<br>• How do you think patients might react to the introduction of the software? |
| Intervention Coherence |
| *Purpose: To understand the extent to which participants understand the technology and how it works.*<br><br>**3. Can you tell me a bit about how the software works and what kind of changes it is expected to bring to standard of care?**<br><br>*Possible follow-up questions:*<br>• How important is it for you to understand the technical details of the software?<br>• How important do you feel it is that patients have a good understanding of how the software works? |

### Burden & Opportunity Costs

*Purpose: To understand the perceived amount of effort that is required to implement the technology and any benefits, profits, or values, that participants feel they need to give up, to engage in the intervention.*

4. **What kind of changes are needed for the implementation of the software? What kind of changes do you personally need to do?**

   *Possible follow-up questions:*

- How easy do you think it will be to implement the software?
- What kind of impact do you think it will have on your workload?
- What kind of impact do you think it might have on your relationship with other staff?
- What kind of impact do you think it might have on your relationship with patients?

### Ethicality

*Purpose: To understand the extent to which the technology has good fit with an individual's value system.*

5. **There is a lot of discussion about the ethical and legal challenges of using AI technologies in health care. What is your view on this?**

   *Possible follow-up questions:*

- Do you think these challenges might apply to the implementation of the **qXR** software and, if yes, how could they be addressed?

### Perceived Effectiveness

*Purpose: To understand the extent to which the technology is perceived as likely to achieve its purpose.*

6. **To what extent do you think that the qXR Software is likely to achieve its purpose?**

   *Possible follow-up questions:*

- What are your biggest concerns when it comes to the performance of the system?
- What kind of opportunities do you think might arise from the introduction of the system?

### Self-efficacy

*Purpose: To understand the participants' confidence that they can perform the behavior required to participate in the intervention.*

7. **Overall, how confident do you feel right now in using the qXR software?**

   *Possible follow-up questions:*

- What kind of additional support or information would you like to have?

### Wrap Up

Thank you for taking the time to share your opinions with me today. We truly appreciate your willingness to participate and discuss your thoughts and ideas with us. We've now reached the end of our discussion.

8. **Is there anything else that you would like to add?**

**POST-IMPLEMENTATION STAFF INTERVIEW GUIDE**

**Retrospective Acceptability of the qXR Artificial Intelligence Software**

| Introduction |
| --- |
| Thank you for Thank you for agreeing to talk to me again -I really appreciate your time and effort. Last time you raised several important issues related to the forthcoming introduction of the **qXR** Artificial Intelligence Software. Today, I would like to know more about what your actual experience with the software has been, whether there were things that worked better or worse than you anticipated, and what your views are about facilitators and barriers to scale up.<br><br>Let me remind you once again that all of the feedback you share with me today will remain strictly confidential. Your name will not be attached to any of your comments, and any other potentially identifying information will be deleted or edited out from the transcriptions, to make sure that what you tell us remains private. Shall I start the recording? Are you happy to get started? |
| Affective Attitude |
| *Purpose: To understand how participants feel about the technology.*<br><br>**1. What are your thoughts about the qXR Artificial Intelligence Software now that you have some experience with it?**<br><br>*Possible follow-up questions:*<br>• How do you think your colleagues felt about the introduction of the software?<br>• How did patients react to the introduction of the software? |
| Intervention Coherence |
| *Purpose: To understand the extent to which participants understand the technology and how it works.*<br><br>**2. Can you tell me a bit about how the software worked and what kind of changes it brought to standard of care?**<br><br>*Possible follow-up questions:*<br>• Do you feel you know more about the technical details of the software?<br>• Do you think patients understood how the software worked? |
| Burden & Opportunity Costs |
| *Purpose: To understand the perceived amount of effort that is required to implement the technology and any benefits, profits, or values, that participants feel they need to give up, to engage in the intervention.*<br><br>**3. What kind of changes were made for the implementation of the software? What kind of changes did you personally do?**<br><br>*Possible follow-up questions:*<br>• How easy was it to implement the software?<br>• What kind of impact did it have on your workload?<br>• How did it impact on your relationship with other staff and patients?<br>• Were there things that worked better or worse than you anticipated? |
| Ethicality |

*Purpose: To understand the extent to which the technology has good fit with an individual's value system.*

4. **Last time you talked about the ethical and legal challenges of using AI technologies in health care. What are your views on this now?**

   <u>*Possible follow-up questions:*</u>

- Did these challenges apply to the implementation of the **qXR** software and, if yes, how were they addressed?

**Perceived Effectiveness**

*Purpose: To understand the extent to which the technology is perceived as likely to achieve its purpose.*

5. **To what extent do you think that the qXR Software achieved its purpose?**

   <u>*Possible follow-up questions:*</u>

- What kind of opportunities do you think might arise from the broader implementation of the system? And what do you think the biggest challenges will be?
- Based on your experience, what factors might facilitate successful adoption and scaling up of the technology? What might hinder it?

**Self-efficacy**

*Purpose: To understand the participants' confidence that they can perform the behavior required to participate in the intervention.*

6. **Overall, how confident do you feel right now in using the qXR software?**

   <u>*Possible follow-up questions:*</u>

- Do you feel you got all of the support and information needed to implement the software?

**Wrap Up**

Thank you for taking the time to share your opinions with me today. We truly appreciate your willingness to participate and discuss your thoughts and ideas with us. We've now reached the end of our discussion.

7. **Is there anything else that you would like to add?**

**PATIENT FOCUS GROUP GUIDE**

**Concurrent Acceptability of the qXR Artificial Intelligence Software**

| Introduction & Ice Breaker |
|---|
| Thank you for making the time to talk to me today. I really appreciate your time and commitment. As you know already, we are talking to both patients and staff, to better understand their views about the recent introduction of the qXR Artificial Intelligence Software, which was created to help identify and prioritise patients for further investigation when their chest x-ray is suspicious of cancer. All of the feedback you give me today will remain strictly confidential. You've noticed this already, but just to remind you that this session is being recorded. Your name, however, will not be attached to any of your comments, and any other potentially identifying information will be deleted or edited out from the transcriptions, to make sure that what you tell us remains private. Do you have any questions or is there anything else you would like to know before we start?<br><br>Let me give you a few quick ground rules before we begin. First, I have a list of key questions that I would like to ask you, but you may also have some information or ideas that we haven't thought of. I really want to hear what you think is important about this topic, so please feel free to tell me whatever you think relevant. Everyone's opinion is valued and it's ok to disagree with each other or with me. We are interested in hearing all points of view. It's also fine to talk to each other and not just to me. This is meant to be a discussion rather than a Q&A session. Are you happy to get started?<br><br>**1. Shall we start with a round of introductions? Can everyone give me their first name and a 'fun fact' about themselves?**<br><br>*Moderator starts by sharing a 'fun fact' about himself/herself and then moves on to the next person.* |
| Affective Attitude |
| *Purpose: To understand how participants feel about the technology.*<br><br>**2. The purpose of today's meeting is to discuss your views about the introduction of the qXR Artificial Intelligence Software. What were your first thoughts when you heard about it?**<br><br><u>*Possible follow-up questions:*</u><br>• How were you informed about the introduction of the software?<br>• How clear did you find the information provided?<br>• Did you discuss this with your family?<br>• How did they feel about it? |
| Intervention Coherence |
| *Purpose: To understand the extent to which participants understand the technology and how it works.*<br><br>**3. What is your understanding of the software and how it works?**<br><br><u>*Possible follow-up questions:*</u><br>• How important is it for you to understand the technical details of the software? |

| Burden & Opportunity Costs |
|---|
| *Purpose: To understand the perceived amount of effort that is required to implement the technology and any benefits, profits, or values, that participants feel they need to give up, to engage in the intervention.*<br><br>**4. What are the positive and what are the negative aspects of introducing this AI technology in the NHS?**<br><br><u>*Possible follow-up questions:*</u><br>• What kind of impact do you think the qXR Artificial Intelligence Software had on your care? |
| Ethicality |
| *Purpose: To understand the extent to which the technology has good fit with an individual's value system.*<br><br>**5. There is a lot of discussion about the ethical and legal challenges of using AI technologies in health care. What is your view on this?**<br><br><u>*Possible follow-up questions:*</u><br>• Do you think these challenges might apply to the introduction of the qXR Artificial Intelligence Software, and if yes, how could they be addressed? |
| Perceived Effectiveness |
| *Purpose: To understand the extent to which the technology is perceived as likely to achieve its purpose.*<br><br>**6. To what extent do you think that the qXR Artificial Intelligence Software will be effective in reducing delays to obtaining a CT scan when necessary after chest X-ray?**<br><br><u>*Possible follow-up questions:*</u><br>• Do you have any concerns about the software?<br>• What kind of opportunities do you think might arise from a broader implementation of the software? |
| Wrap Up |
| Thank you for taking the time to share your opinions with me today. We truly appreciate your willingness to participate and discuss your thoughts and ideas with us. We've now reached the end of our discussion.<br><br>**7. Is there anything else that you would like to add?** |
